# Applying time-frequency analysis to assess cerebral autoregulation during hypercapnia

**DOI:** 10.1371/journal.pone.0181851

**Published:** 2017-07-27

**Authors:** Michał M. Placek, Paweł Wachel, D. Robert Iskander, Peter Smielewski, Agnieszka Uryga, Arkadiusz Mielczarek, Tomasz A. Szczepański, Magdalena Kasprowicz

**Affiliations:** 1 Department of Biomedical Engineering, Faculty of Fundamental Problems of Technology, Wroclaw University of Science and Technology, Wroclaw, Poland; 2 Department of Control Systems and Mechatronics, Faculty of Electronics, Wroclaw University of Science and Technology, Wroclaw, Poland; 3 Division of Neurosurgery, Department of Clinical Neurosciences, University of Cambridge, Cambridge, United Kingdom; 4 Department of Cybernetics and Robotics, Faculty of Electronics, Wroclaw University of Science and Technology, Wroclaw, Poland; 5 Department of Neurosurgery, Lower Silesia Specialist Hospital, Wroclaw, Poland; Nanjing Normal University, CHINA

## Abstract

**Objective:**

Classic methods for assessing cerebral autoregulation involve a transfer function analysis performed using the Fourier transform to quantify relationship between fluctuations in arterial blood pressure (ABP) and cerebral blood flow velocity (CBFV). This approach usually assumes the signals and the system to be stationary. Such an presumption is restrictive and may lead to unreliable results. The aim of this study is to present an alternative method that accounts for intrinsic non-stationarity of cerebral autoregulation and the signals used for its assessment.

**Methods:**

Continuous recording of CBFV, ABP, ECG, and end-tidal CO_2_ were performed in 50 young volunteers during normocapnia and hypercapnia. Hypercapnia served as a surrogate of the cerebral autoregulation impairment. Fluctuations in ABP, CBFV, and phase shift between them were tested for stationarity using sphericity based test. The Zhao-Atlas-Marks distribution was utilized to estimate the time—frequency coherence (TFCoh) and phase shift (TFPS) between ABP and CBFV in three frequency ranges: 0.02–0.07 Hz (VLF), 0.07–0.20 Hz (LF), and 0.20–0.35 Hz (HF). TFPS was estimated in regions locally validated by statistically justified value of TFCoh. The comparison of TFPS with spectral phase shift determined using transfer function approach was performed.

**Results:**

The hypothesis of stationarity for ABP and CBFV fluctuations and the phase shift was rejected. Reduced TFPS was associated with hypercapnia in the VLF and the LF but not in the HF. Spectral phase shift was also decreased during hypercapnia in the VLF and the LF but increased in the HF. Time-frequency method led to lower dispersion of phase estimates than the spectral method, mainly during normocapnia in the VLF and the LF.

**Conclusion:**

The time—frequency method performed no worse than the classic one and yet may offer benefits from lower dispersion of phase shift as well as a more in-depth insight into the dynamic nature of cerebral autoregulation.

## Introduction

Cerebral autoregulation (CA) is an intrinsic control mechanism stabilizing cerebral blood flow (CBF) supply in the face of moderate changes in arterial blood pressure (ABP). In early studies, the pressure-flow relationship was described as a nonlinear curve with the flat part indicating a working range of CA [1]. Such an approach led to a view that CA maintains constant CBF despite changes of ABP and that CA is a static phenomenon. With introduction of transcranial Doppler ultrasonography (TCD), which allows non-invasive measurement of cerebral blood flow velocity (CBFV) with high temporal resolution, it has been demonstrated that CBFV can rapidly vary in response to sudden changes in ABP. Since then, an assessment of dynamic properties of CA has been performed by investigating the response of CBFV following either spontaneous fluctuation of ABP [2] or a manipulation in ABP induced by the interventions, such as: the thigh cuff deflation [3], Valsalva maneuver [4], the postural change from sitting to standing [5] or squatting to standing [6]. Dynamic CA is often investigated during hypercapnia [7]–a state of elevated arterial CO_2_ concentration (typically assessed using end-tidal CO_2_ measurement—EtCO_2_) above its normal level (normocapnia). It leads to dilatation of small cerebral arteries and arterioles and to a subsequent increase of CBFV. This vasodilatation, if sufficiently strong, severely reduces capacity of the autoregulatory mechanism and thus is often used to simulate pathological impairment of CA [8].

CA can be impaired in patients suffering from life treating diseases, such as traumatic brain injury, subarachnoid hemorrhage, or stroke. It is important for diagnosis and management of such patients to assess properly the effectiveness of CA and describe in detail the interactions between oscillations in ABP and CBFV.

A popular method for assessing dynamic CA is a transfer function analysis performed using the Fourier transform to quantify the relationship between ABP and CBFV [9]. This method results in the spectral coherence function and the phase frequency response (and/or amplitude ‘gain’ frequency response). These parameters have physical meaning and are easy to interpret. Increased values of coherence (and gain) and lower values of phase shift are considered as indicators of CA worsening and vice-versa. It is worth noticing that the use of coherence function to study dynamic CA serves two purposes that are contradictory [10]. First, coherence is treated as a measure of coupling between the input (ABP) and the output (CBFV). It has been postulated that working CA should lead to uncoupling between ABP and CBFV and hence to low value of coherence in healthy subjects (particularly with the low frequency band where dynamic CA is expected to be the most efficient [10]), whereas in patients with impaired CA changes in ABP might be passively followed by changes in CBFV, therefore the value of coherence should be high [11]. However, a low value of coherence might be also a consequence of nonlinear input-output relationship, co-variants that were not accounted for, or noise [12–14]. Therefore, coherence can be low, even if CA is greatly impaired. Because the source of low coherence is difficult to identify, the phase shift is considered more promising index of CA than coherence [15], but a criterion of high value of coherence may be used to safeguard validity of phase estimation between CBFV and ABP [16–18].

The transfer function method has proved to be useful in the clinical environments [19–23], but it has limitations. A restriction of the spectral studies is that they usually assume signals to be stationary (although this is not a strict requirement), whereas fluctuations of both ABP and CBFV, either spontaneous or evoked by the hemodynamic maneuvers, are transient and inherently non-stationary. The traditional techniques based on Fourier transform may arrive at non-unique spectral representations of these non-stationary signals. Furthermore, spectral estimation of phase shift is only accurate when corresponding coherence is high. Low spectral coherence is, however, often observed in clinical and experimental settings, particularly in the low frequency band [13,14,24]. This has encouraged some attempts to use advanced nonlinear system identification techniques for CA including high-order Volterra series [25–27]. Even though nonlinear components contribute to improve model quality, it is difficult to gain physical insights from this nonparametric approach [24,28]. Moreover, increasing accuracy of Volterra models usually requires exponential growth of number of necessary Volterra kernels. It has also been suggested that significantly higher influence on coherence reduction, compared to the non-linear effects, can be attributed to an extra input to the system, namely arterial tension of CO_2_ [10,28]. Multivariate coherence that measures extent to which CBFV can be represented as a linear system of multiple inputs was showed to be significantly higher than univariate coherence [28]. Although this finding demonstrates that coherence in low frequency range is more effectively modeled as a multiple-input system and the low value of coherence may be, at least partially, due to the effects of CO_2_ changes on CBFV variability, it does not address the issue of optimal estimation of the phase shift between two determinants of CBFV changes. Furthermore, since CA is considered to be a non-stationary mechanism [15], the strength of coherence function between fluctuations in ABP and CBFV can change in time as well, which warrants application of time-varying identification techniques.

In this work we aimed to describe the new approach for the ABP-CBFV relationship analysis using joint time-frequency (T-F) method. This approach overcomes the restrictions intrinsic to the stationary conditions and allows for solid estimation of phase shift between fluctuations in ABP and CBFV in T-F regions where linear model is locally validated by statistically justified value of magnitude-squared time-frequency coherence (TFCoh). We estimated the CA parameters in non-stationary conditions and compared them with the classic spectral transfer function estimates of CA.

## Material and methods

### Subjects, experimental procedure & data acquisition

Fifty volunteers (29 females and 21 males, median age: 23, range: 18–31 years) were recruited from student’s community of the Wroclaw University of Technology via advertisement posted on the university website. Medical history review and physical examination were performed by an experienced physician (TAS). Exclusion criteria included smokers, hypertension, systemic diseases, cardiovascular and neurological disorders. Subjects were not on any medication known to alter cardiovascular parameters. Subjects were asked to refrain from alcohol or caffeine consumption for at least 12 hours before the examination. Studies were performed at room temperature with the supervision of physician (TAS). All volunteers provided written informed consent before participation and were treated in accordance with the Declaration of Helsinki. Ethical approval was obtained from the Commission of Bioethics at Wroclaw Medical University, Wroclaw, Poland (permission no. KB– 170/2014), before commencing the study.

**Fig 1:**
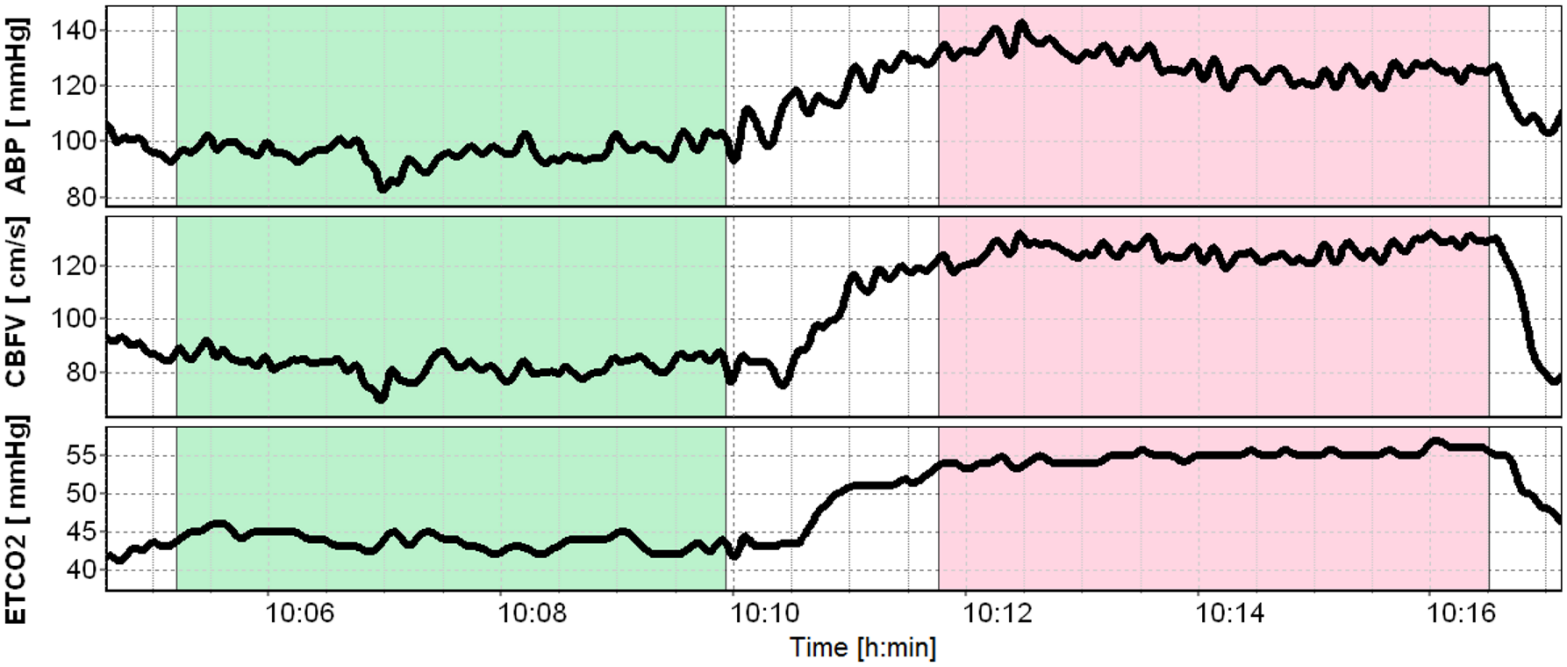
Exemplary smoothed time courses (average filter) of physiological signals. ABP—arterial blood pressure, CBFV—cerebral blood flow velocity, EtCO_2_ –end-tidal CO_2_.

All volunteers were given at least 15 minutes to relax before measurement. First, the experimental data were collected at rest, under natural breathing condition. After about 5 minutes of baseline recording EtCO_2_ concentration was raised by attaching a plastic tube to the subject’s face mask, thereby increasing the respiratory dead space by 1.5 liter. After reaching an EtCO_2_ plateau for 5 min (hypercapnia), the tube was removed and the subjects were asked to breath naturally (Fig 1).

CBFV in the left middle cerebral artery was recorded using transcranial Doppler ultrasonography (TCD) (Doppler-Box^™^ X DWL, Singen, Germany) with 2 MHz probe attached to a head frame. ABP was measured noninvasively using volume-clamping method (Finometer^®^ MIDI, FMS Medical Systems, Amsterdam, Netherlands) via a cuff placed around the middle finger of the left hand held at the level of the heart. A three lead ECG (ECG module, FMS Medical Systems, Amsterdam, Netherlands) was used for measurement of cardiac activity. A breathing mask was applied to the patient’s nose and mouth and connected to a standard capnograph (RespSense^™^, NONIN, Plymouth, MA, USA) for EtCO_2_ as well as respiratory rate continuous measurement. Raw data were captured with 200 Hz sampling frequency using ICM+^®^ (Cambridge Enterprise Ltd, Cambridge, UK) and further processed with custom written program in MATLAB^®^ (MathWorks^®^, Natick, MA, USA).

### Signal analysis

First, two segments of data recorded during normocapnia and hypercapnia (plateau phase) were extracted from each monitoring session (see Fig 1). Raw ABP and CBFV data were subsequently smoothed and down-sampled. We used the Pan-Tompkins algorithm [29] to detect QRS complexes within the ECG signal. Raw ABP and CBFV were then averaged within short intervals delimited by the instants of R peaks in ECG. These intervals had different length due to the variable heart rate. Next, equidistant samples corresponding to the sampling frequency of 2 Hz were obtained using cubic spline interpolation. Eight leading and eight trailing samples were discarded to diminish boundary effects caused by interpolation. Resampled data segments were then detrended separately for normocapnia and hypercapnia by subtracting the regression line (c.f. [13]). After performing T-F analysis, we extracted three frequency ranges: 0.02–0.07 Hz (very low frequency range, VLF), 0.07–0.20 Hz (low frequency range, LF), and 0.20–0.35 Hz (high frequency range, HF), where CA is thought to be operating [14,30].

#### Assessment of non-stationarity

ABP and CBFV signals were tested for stationarity using sphericity based test [31]. Accordingly, the considered signal *x* was divided into *M* non-overlapping segments of equal lengths. For each segment *m* = 1, …, *M* power spectrum $S_{xx}^{m}\left(\omega \right)$ was calculated. The null hypothesis of stationarity *H*_0_ at a frequency *ω*_*k*_ versus the alternative of non-stationarity was defined as:

$$H_{0}:\;\;\text{log}\frac{\frac{1}{M}\sum_{m=1}^{M}S_{xx}^{m}\left(\omega_{k}\right)}{\sqrt[{M}]{\prod_{m=1}^{M}S_{xx}^{m}\left(\omega_{k}\right)}}=0.$$ (1)

The above test statistics is equal to zero if, and only if, $S_{xx}^{m}\left(\omega_{k}\right)$ are identical in consecutive segments, which implies stationarity at frequency *ω*_*k*_. In practice *ω*_*k*_ represents evenly spaced frequency bins. In our case, four bins were examined, namely: 0–0.1 Hz, 0.1–0.2 Hz, 0.2–0.3 Hz, and 0.3–0.4 Hz. A signal was considered as nonstationary, if *H*_0_ was rejected at a significance level of 0.0125 for at least one frequency bin. Critical values used to reject the hypothesis of stationarity were estimated empirically in the way of Monte Carlo simulations [31].

In addition, the phase shift between ABP and CBFV was also tested for stationarity. To do so, sphericity based test was adjusted in the following way. Power spectrum $S_{xx}^{m}\left(\omega \right)$ was replaced by phase spectrum $\left(\text{arg}\;S_{xy}^{m}\left(\omega \right)\right)$. The range of argument was expressed as [0,2*π*), instead of (−*π*, *π*], because the original test, based on power spectra, assumes that spectra are nonnegative. Critical values used to reject the hypothesis of stationarity of phase at a given significance level were established in the way of Monte Carlo simulations (10,000 repetitions), similarly to the original test. White Gaussian noise was generated as the first signal, which was then high-pass filtered giving the second signal. Generated signals should not be uncorrelated, since then their estimated coherence is close to zero and the phase shift becomes meaningless. To resemble real data, high-pass filter was chosen to correlate simulated signals because it models dynamic pressure—flow relationship.

#### Time-frequency representation

**Fig 2:**
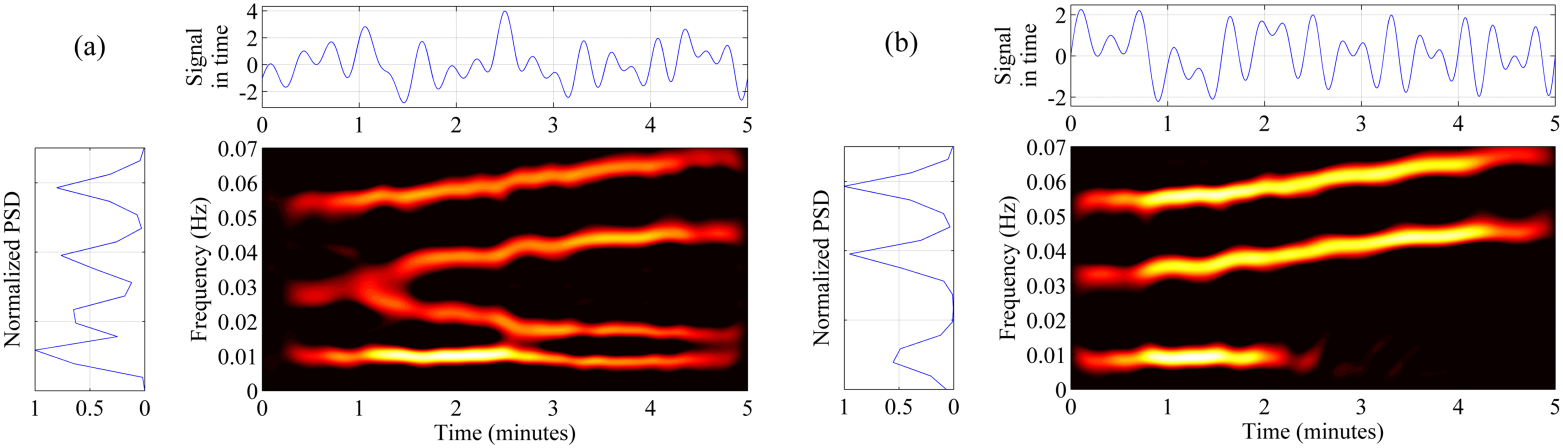
Calibration signals. Time (top subplot), frequency (left subplot), and time—frequency (center graph) representations of the calibration signals consisting of four components (a) and three components (b).

For the purpose of T-F representation (TFR) evaluation we chose Zhao-Atlas-Marks distribution (ZAMD) [32] for its relatively high suppression of the cross-terms. Implementation available in [33] allows to employ two windows, in contrast to the original ZAMD which uses only frequency smoothing window. Cross-ZAMD can be described as follows:

$$S_{ZAM,xy}\left(t,f\right)=\int_{-\infty }^{\infty }h\left(\tau \right)e^{-j2\pi f\tau }\left[\int_{t-\left|\tau \right|/2}^{t+\left|\tau \right|/2}g\left(u-t\right)x\left(u+\frac{\tau }{2}\right)y^{*}\left(u-\frac{\tau }{2}\right)du\right]d\tau$$ (2)

where *h* and *g* are window functions (chosen here as Hanning). The longer the window *g*, the stronger the smoothing is in the time domain, whereas the longer window *h*, the weaker the smoothing is in the frequency domain. Signals *x* and *y* are analytic, i.e., complex signals created by adding to real-valued signal its Hilbert transform multiplied by the imaginary unit *j*. By *S*_*ZAM*,*xx*_ (*t*, *f*) and *S*_*ZAM*,*yy*_ (*t*, *f*) we denote the auto-ZAMD for signals *x* and *y*, respectively. Since the size of both windows has an essential influence on the resulting TFR we tuned the length of *h* and *g* in the following numerical experiment. We constructed a test data resembling smoothed ABP and CBFV signals. To this end we generated time series consisted of 0.01 Hz sinusoid and three frequency-modulated signals with frequencies changing linearly within 5 minutes from 0.03, 0.03, 0.05 to 0.01, 0.05, 0.07 Hz, respectively (Fig 2a) [34]. Such calibration data is representative for higher frequencies as well, since ZAMD has the property of *time and frequency covariance* [33], i.e. if $z\left(t\right)=x\left(t-t_{0}\right)e^{j2\pi f_{0}t}$, then *S*_*ZAM*,*zz*_ (*t*, *f*) = = *S*_*ZAM*,*xx*_ (*t* − *t*_0_, *f* − *f*_0_). This property guarantees that, if the signal is modulated, its time-frequency distribution is simply translated in the frequency domain. For proper selection of the lengths of windows (that provide good trade-off between satisfactory T-F resolution and strong reduction of interferences), we introduced a criterion Q consisting of two additive components

$$Q\left(L_{h},L_{g}\right)=REP+IP$$ (3)

where REP stands for Rényi entropy penalty and IP is the interference penalty. We utilized REP to optimize resolution of TFR [35], whereas IP was used to control presence of interferences. For each time instant in TFR, local maxima in frequency slices were counted and their average was taken as IP. The lengths of windows *L*_*h*_ = 449 samples and *L*_*g*_ = 513 that minimized the above-mentioned criterion were considered as close to the optimal and applied in further calculations.

#### Time-frequency coherence

Non-stationary signals cannot be properly studied using pure spectral coherence, since the temporal information is lost in that approach. This limitation can be overcome by replacing classic Fourier-transform-based spectra in coherence definition with time-varying spectra.

TFCoh for signals *x* and *y* is defined as follows: [36]

$${\left|C_{xy}\left(t,f\right)\right|}^{2}=\frac{{\left|S_{xy}\left(t,f\right)\right|}^{2}}{S_{xx}\left(t,f\right)\;\cdot \;S_{yy}\left(t,f\right)},\;\;\left(t,f\right)\in R_{x}\cap R_{y}$$ (4)

where *S*_*xy*_ stands for cross spectrum, *S*_*xx*_, *S*_*yy*_ are auto spectra, and *R*_*x*_, *R*_*y*_ denote the regions in (*t*, *f*) plane where *S*_*xx*_ > 0 and *S*_*yy*_ > 0, respectively. Thus, *C*_*xy*_(*t*, *f*) can be defined only for those (*t*, *f*) points in time-frequency plane where auto spectra are positive.

In this paper, ZAMD (being a member of Cohen’s class) was used to estimate TFCoh [37]. To meet the requirement that |*C*_*xy*_(*t*, *f*)| is bounded by one, additional smoothing of TFR should be performed [38]. In effect TFCoh estimator employing smoothed ZAMD is defined as follows: [37]

$${\left|{\hat{C}}_{ZAM,xy}^{\left(c\right)}\left(t,f\right)\right|}^{2}=\frac{{\left|{\hat{S}}_{ZAM,xy}^{\left(c\right)}\left(t,f\right)\right|}^{2}}{{\hat{S}}_{ZAM,xx}^{\left(c\right)}\left(t,f\right)\;\cdot \;{\hat{S}}_{ZAM,yy}^{\left(c\right)}\left(t,f\right)},\left(t,f\right)\in R_{x}\cap R_{y}$$ (5)

where ${\hat{S}}_{ZAM,xy}^{\left(c\right)}\left(t,f\right)={\hat{S}}_{ZAM,xy}\left(t,f\right)**c\left(t,f\right)$. Operator ** indicates 2-D convolution, and *c*(*t*, *f*) is a smoothing kernel, chosen here as a Gaussian function with *σ*_*t*_ = 10 seconds and *σ*_*f*_ = 0.0004 Hz empirically chosen such that both time and frequency support are maintained. In order to avoid computational singularities we assumed coherence equal to zero when auto spectra in Eq (5) were non-positive, despite additional smoothing. Whenever we refer to the TFCoh value, we understand by it the magnitude-squared coherence. In the following, to simplify the notation, we will write ${\left|\hat{C}\right|}^{2}={\left|{\hat{C}}_{ZAM,xy}^{\left(c\right)}\left(t,f\right)\right|}^{2}$.

**Fig 3:**
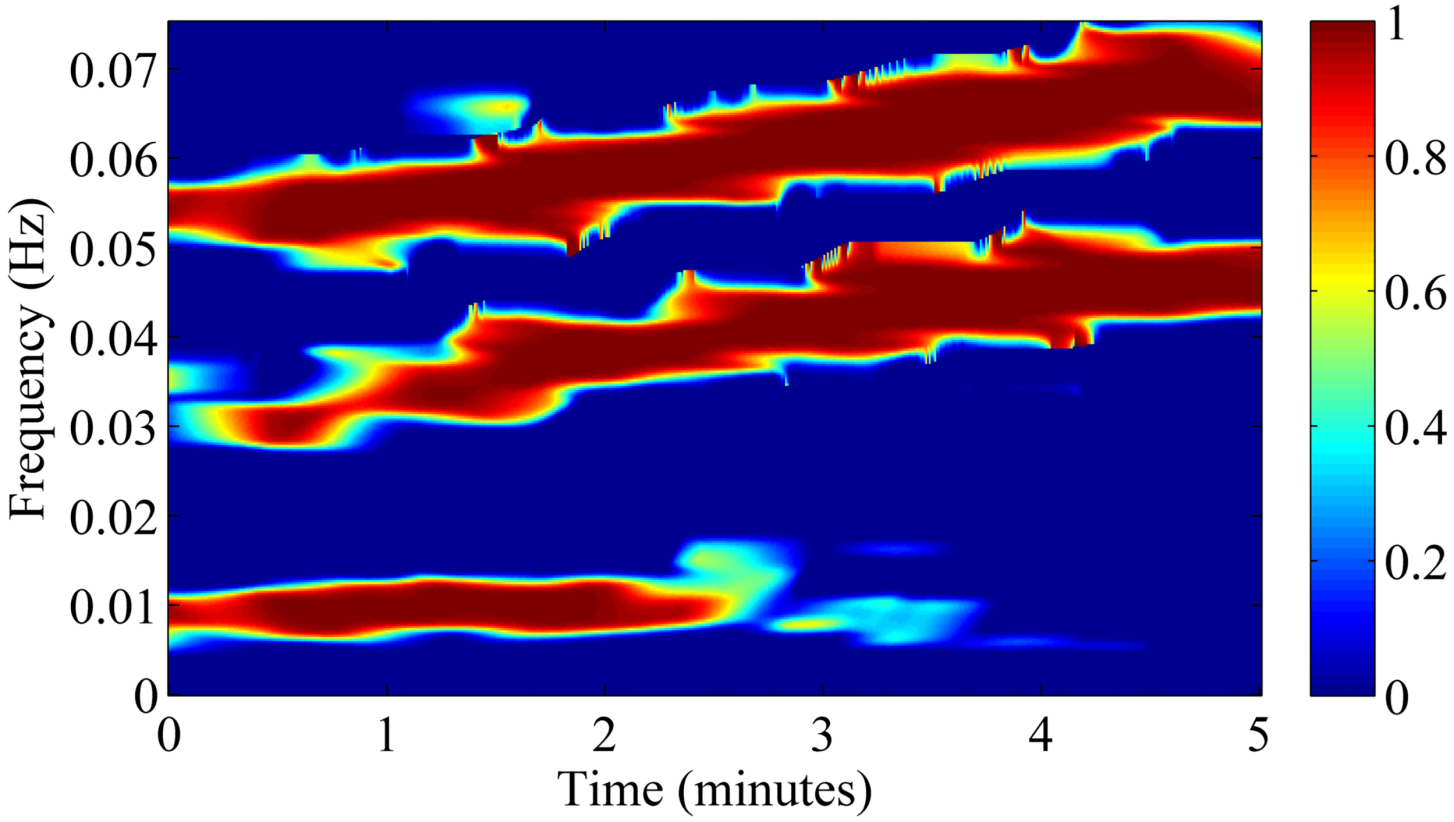
Time-frequency magnitude-squared coherence between the two signals presented in Fig 2. Coherence was estimated using Eq (5). It can be seen that coherence is high in the regions where both signals had components in their time-frequency representations.

To test the TFCoh estimator, we generated another signal (Fig 2b) which consisted of three components common with the signal presented in Fig 2a (third component was common only for *t* < 3 min). TFCoh revealed coupling between signals from Fig 2 only in the T-F area of common components (Fig 3).

**Fig 4:**
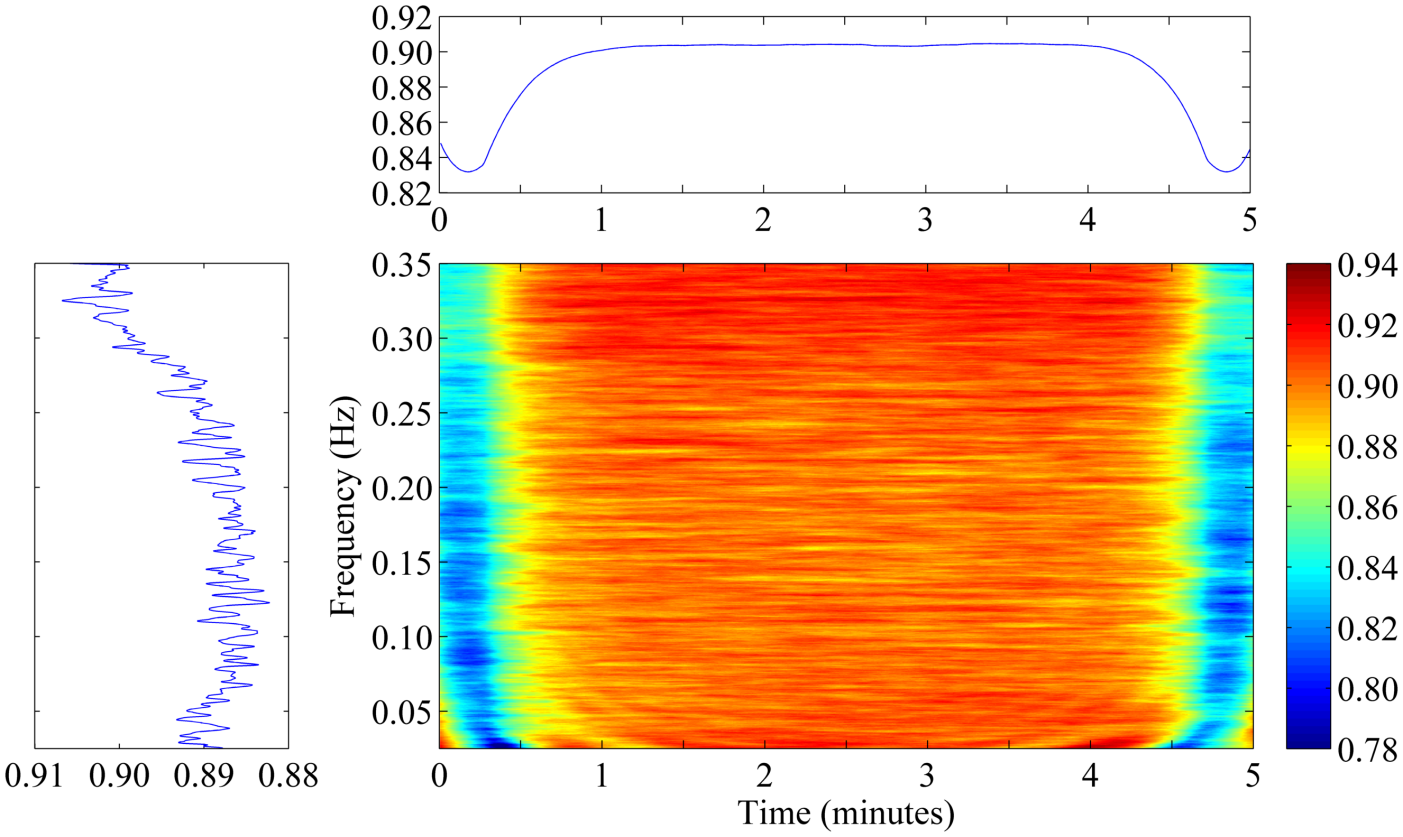
The 95^th^ percentile for the time-frequency coherence function established on the basis of Monte Carlo simulations. Center graph shows the 95^th^ percentile of the statistical distribution consisting of 10,000 realizations of time-frequency coherence between band-pass filtered Gaussian noises. Left and top subplots show the average of that percentile with respect to time and frequency, respectively.

Following the approach presented in [39,40], the threshold parameter *T*_*h*_ for significant coherence was tuned on the basis of Monte Carlo simulations. In each of 10,000 repetitions, a pair of 5-minutes-long, band-pass filtered Gaussian noise signals were generated and their mutual TFCoh(*t*, *f*) was evaluated. Next, the 95^th^ percentile of TFCoh(*t*, *f*) was calculated for each point of considered T-F area (*t* < 300 s, 0.02 < *f* < 0.35 Hz). The result is presented in Fig 4 (center graph shows the 95^th^ percentile in time-frequency plane, while left and top subplots show the average of that percentile with respect to time and frequency, respectively). It can be seen that the 95^th^ percentile was slightly lower at the beginning and at the end of the recording, what can be attributed to the boundary effect. Apart from that area, the percentile is approximately equal to 0.9; therefore, we took value *T*_*h*_ = 0.9 as a resulting threshold for significant coherence. This threshold was used for selecting T-F regions to estimate phase shift within (described in next section).

In order to assess T-F coupling between oscillations in ABP and CBFV we considered two metrics based on TFCoh. Average magnitude-squared time-frequency coherence (aTFCoh) is simply average value of TFCoh in T-F domain.

$$\text{aTFCoh}={\text{average}}_{t\in T,f\in B}\left({\left|\hat{C}\right|}^{2}\right)$$ (6)

In Eq (6) *T* denotes the duration of signals *x* and *y*, whereas *B* stands for investigated frequency range. In addition, we calculated the ratio (expressed in %) of the areas where the value of TFCoh was above the threshold *T*_*h*_ to the total area of T-F plane (in the investigated frequency range; i.e. VLF, LF, or HF).

#### Time—frequency phase shift

We employed T-F analysis to calculate time-frequency phase shift (TFPS) between oscillations in ABP and CBFV. TFPS was derived from the cross phase spectrum Θ_*xy*_ (*t*, *f*) which was calculated as the argument of the cross-spectral density *S*_*xy*_(*t*, *f*). [40]

$$\Theta_{xy}\left(t,f\right)=arg\left[S_{xy}\left(t,f\right)\right],\;\;\;\left(t,f\right)\in R_{x}\cap R_{y}\cap {\mathbb{R}}^{2}:{\left|\hat{C}\right|}^{2}\geq T_{h}$$ (7)

Estimated values of TFPS between signals *x* and *y* are meaningful only for those (*t*, *f*) points in T-F plane where TFCoh between *x* and *y* is significantly high. Only values above the *T*_*h*_ = 0.9 were considered significant (see previous section).

In terms of CA, TFPS between ABP and CBFV, expressed as Θ_*CBFV*,*ABP*_, is positive when CBFV leads ABP [18]. TFPS derived from Eq (7) is bounded by definition to the interval (−*π*, *π*], and ambiguities may occur in interpreting it since real phase difference between two signals can be outside the range (−*π*, *π*].

**Fig 5:**
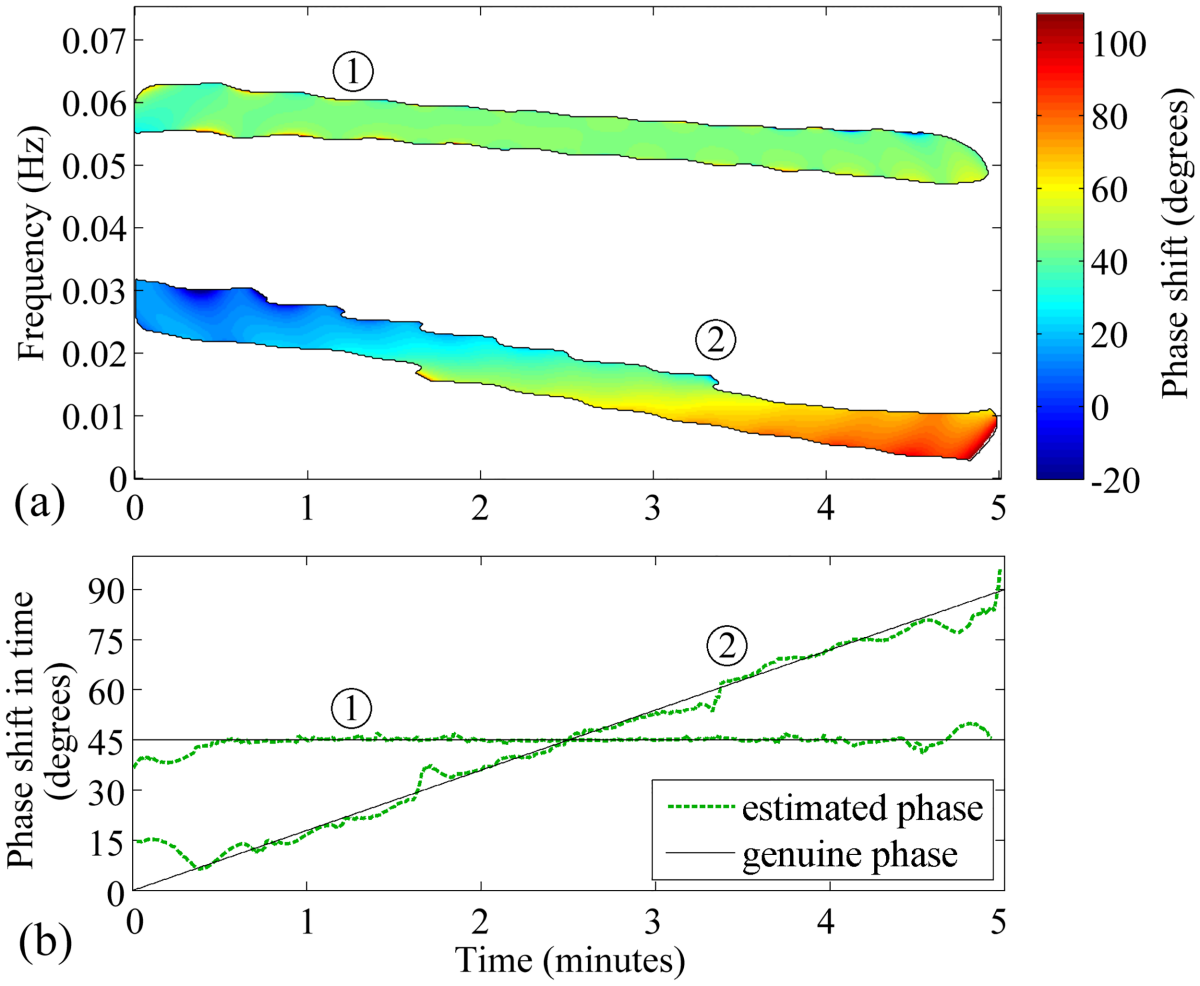
Time-frequency phase shift between two generated signals having two common components. Numbers 1 and 2 surrounded by circles indicate two considered components—frequency-modulated chirps changing linearly within five minutes from 0.06 and 0.03, to 0.05 and 0.003 Hz, respectively. (a) Time-frequency phase spectrum Θ_*xy*_ (*t*, *f*) estimated using Eq (7). Black contour confines area where magnitude-squared coherence was at least 0.9. Remaining area is blank. (b) Time courses of phase shift in two components reconstructed by averaging along the frequency dimension (dashed green lines) and genuine values of the phase (solid black lines).

To examine the ability of Eq (7) in the extraction of TFPS, the following simulation was performed. We generated two signals *x* = *x*_1_ + *x*_2_ and *y* = *y*_1_ + *y*_2_. Components denoted by the same index were localized in the same T-F regions for both *x* and *y*. *x*_1_(*t*) and *x*_2_(*t*) were frequency-modulated signals changing linearly within 5 minutes from 0.06 and 0.03, to 0.05 and 0.003 Hz, respectively. *y*(*t*) = *x*_1_ (*t*)exp(*jθ*_1_) + *x*_2_ (*t*)exp(*jθ*_2_(*t*)), where *θ*_1_ = *π*⁄4, and *θ*_2_ (*t*) = *π* ⋅ *t*[*s*]⁄600. In other words, TFPS between *x*_1_ and *y*_1_ was 45°, while TFPS between *x*_2_ and *y*_2_ was changing linearly from 0° to 90° within 5 minutes. Fig 5a shows TFPS estimated using Eq (7). To reconstruct *θ*_1_ and *θ*_2_(*t*), Θ_*xy*_(*t*, *f*) was averaged along the frequency dimension independently for two components, producing time courses ${\hat{\theta }}_{1}\left(t\right)$ and ${\hat{\theta }}_{2}\left(t\right)$ (Fig 5b). Root mean square errors of ${\hat{\theta }}_{1}\left(t\right)$ and ${\hat{\theta }}_{2}\left(t\right)$ were 2.0° and 3.7°, respectively, and were caused mainly by the boundary effect. Thus, the presented method for TFPS estimation was deemed satisfactory.

In order to assess CA we considered average TFPS (aTFPS) which was calculated as an average over such (*t*, *f*) points where Θ_*xy*_(*t*, *f*) was defined.

#### Spectral phase shift

For the sake of comparison, transfer function derived spectral phase shift (SPS) between oscillations in recorded signals of ABP and CBFV was evaluated separately in each range (i.e., VLF, LF, and HF). SPS as well as magnitude-squared spectral coherence (SCoh) was calculated using the Welch's overlapped averaged periodogram method [41] (five data segments, 100-seconds long Hanning window). Additionally, auto- and cross- spectra were smoothed by convolving them with the window (¼, ½, ¼) as recommended in [42]. Next, in each of three ranges, the frequency of maximal value of SCoh was sought, and representative SPS was calculated for that frequency [43,44]. The value of significant transfer-function derived coherence was established at the level of 0.38 in the way of Monte Carlo simulations, like it was done for TFCoh. We also computed average magnitude-squared spectral coherence (aSCoh) in each frequency range and counted the number of cases when it is below the threshold of 0.38.

### Statistical analysis

Wilcoxon signed-rank test was used to assess the differences between ABP, CBFV, EtCO_2_, as well as SPS and the T-F metrics of dynamic CA in each frequency range during normocapnia and hypercapnia. A value of *p* < 0.05 was considered significant. Nonparametric Friedman ANOVA and the Wilcoxon signed-rank post-hoc comparison test with a Bonferroni adjustment were used to evaluate the differences in aTFCoh, percentage of T-F area at two coherence levels, aTFPS between VLF, LF, and HF ranges. The Bonferroni correction for multiple comparisons was used with a significance level set at α_post-hoc_ < 0.017. Data are reported as medians and interquartile ranges. Pitman-Morgan test was employed to compare variances of T-F estimates of phase shift with spectral ones, and a value of *p* < 0.05 was considered significant.

## Results

The application of the test [31] rejected the hypothesis of stationarity for the majority of ABP, CBFV, and phase shift between them in normocapnia as well as in hypercapnia (see Table 1).

**Table 1:** Rejection rates for testing stationarity.

State	ABP	CBFV	Phase shift
Normocapnia	66%	58%	78%
Hypercapnia	52%	70%	66%

ABP—arterial blood pressure, CBFV—cerebral blood flow velocity.

Hypercapnia caused a significant increase in EtCO_2_, CBFV, as well as ABP (see Table 2). EtCO_2_ was raised by 30.5% [13.5%], CBFV by 28.4% [10.8%], and ABP by 10.6% [12.8%] (median [IQR]). On the other hand, respiratory rate was decreased by 16.4% [22.4%].

**Table 2:** Comparison of changes normocapnia vs. hypercapnia in the values of measured physiological signals.

Parameter	Normocapnia	Hypercapnia	*p*-value
ABP [mm Hg]	88.2 [18.3]	98.3 [20.9]	< 10^−8^
CBFV [cm/s]	71.7 [14.8]	90.3 [25.4]	< 10^−9^
EtCO_2_ [mm Hg]	36.6 [6.1]	47.7 [4.9]	< 10^−9^
RR [breaths / min]	15.2 [5.0]	12.5 [3.5]	< 10^−4^

Values are: median [IQR]. ABP—arterial blood pressure, CBFV—cerebral blood flow velocity, EtCO2 –end tidal CO_2_, RR—respiratory rate.

### Time-frequency coherence results

**Fig 6:**
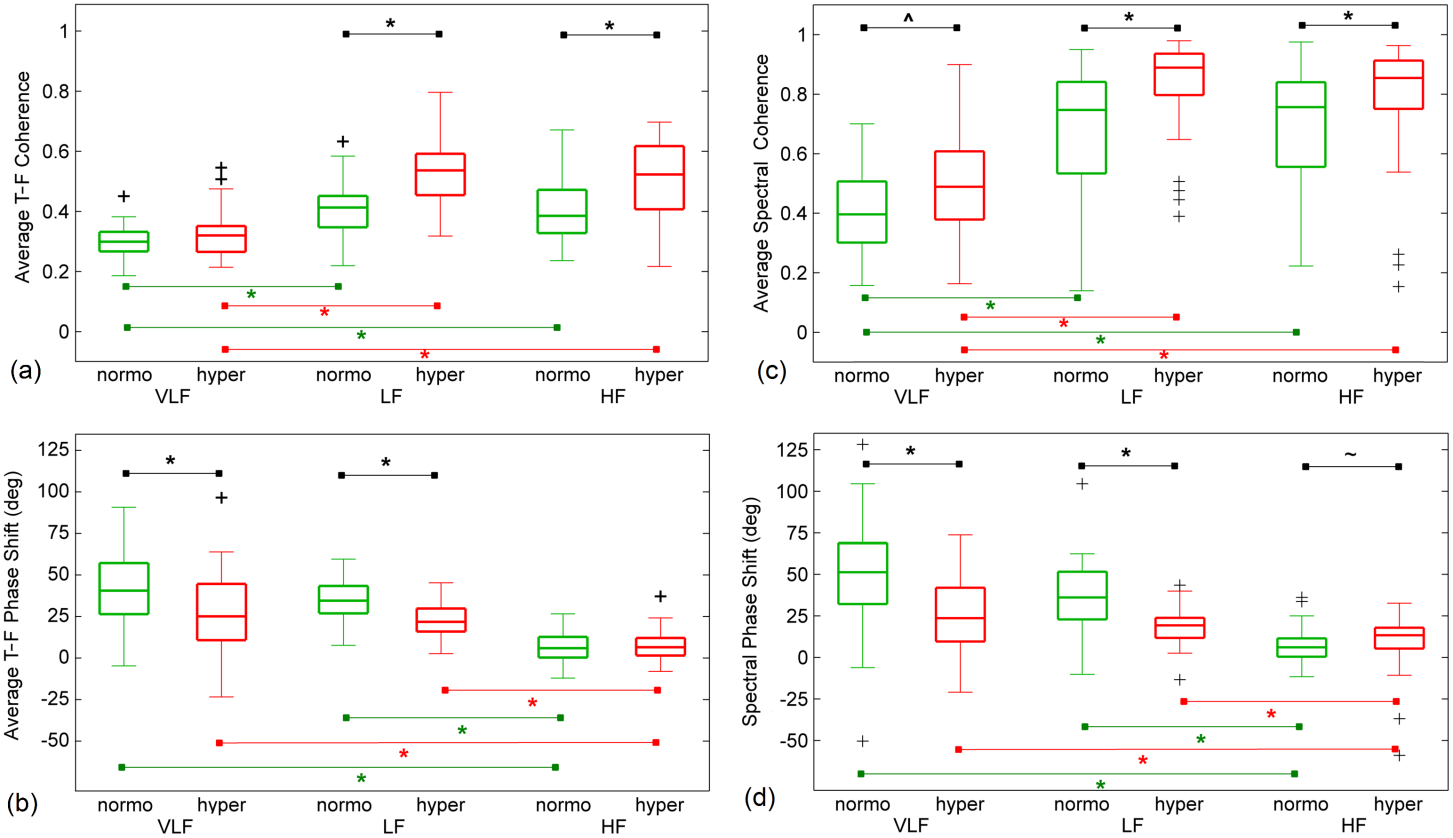
Comparison of the values of selected metrics in normocapnia and hypercapnia in particular frequency ranges. VLF– 0.02–0.07 Hz, LF– 0.07–0.20 Hz, HF– 0.20–0.35 Hz. Outliers defined as values lower than Q1–1.5∙IQR or greater than Q3 + 1.5∙IQR are marked by ‘+’. On each box, the central line is the median, the edges are Q1 and Q3. The whiskers indicate the most extreme data points excluding outliers. Horizontal lines indicate comparison by means of Wilcoxon signed-rank test and corresponding *p*-values are denoted (^~^–*p* < 0.05, ^–*p* < 0.01, *–*p* < 0.001). (a) Average time-frequency coherence, (b) average time-frequency phase shift, (c) average spectral coherence, (d) spectral phase shift estimated at maximal value of spectral coherence within corresponding frequency range.

Table 3 gives the median values and the corresponding interquartile range of T-F coherence metrics during normocapnia and hypercapnia in the VLF, LF, and HF ranges. The aTFCoh was significantly higher in hypercapnia than in normocapnia for all frequency ranges, except for an increase in the VLF that did not reach the level of significance (*p* = 0.063)–see Fig 6a. In the VLF, the percentage of area at high coherence (> 0.9) did not change during hypercapnia when compared with normocapnia. In higher frequency ranges the percentage of area at TFCoh > 0.9 significantly increased during hypercapnia when compared with normocapnia.

**Table 3:** Comparison of changes in the coherence metrics during normocapnia and hypercapnia.

Parameter	Normocapnia	Hypercapnia	*p*-value
**VLF**			
aTFCoh	0.30 [0.06]	0.32 [0.09]	= 0.063
Area of TFCoh > 0.9 (%)	9.39 [5.04]	8.55 [6.46]	= 0.59
**LF**			
aTFCoh	0.41 [0.10]	0.54 [0.14]	< 10^−7^
Area of TFCoh > 0.9 (%)	14.30 [8.58]	23.05 [13.58]	< 10^−7^
**HF**			
aTFCoh	0.39 [0.14]	0.52 [0.21]	< 10^−5^
Area of TFCoh > 0.9 (%)	11.69 [8.41]	18.63 [14.73]	< 10^−4^

Results are: median [IQR]. aTFCoh—average time-frequency coherence. Frequency ranges: VLF– 0.02–0.07 Hz, LF– 0.07–0.20 Hz, HF– 0.20–0.35 Hz.

Comparison of the TFCoh metrics between different frequency ranges revealed significant differences for all parameters. Post-hoc comparisons, however, demonstrated significant differences in both the LF and the HF when compared with the VLF but such a difference was not found between LF and HF ranges. The differences in aTFCoh between different frequency ranges are presented in Fig 6a.

### Time-frequency phase shift results

**Fig 7:**
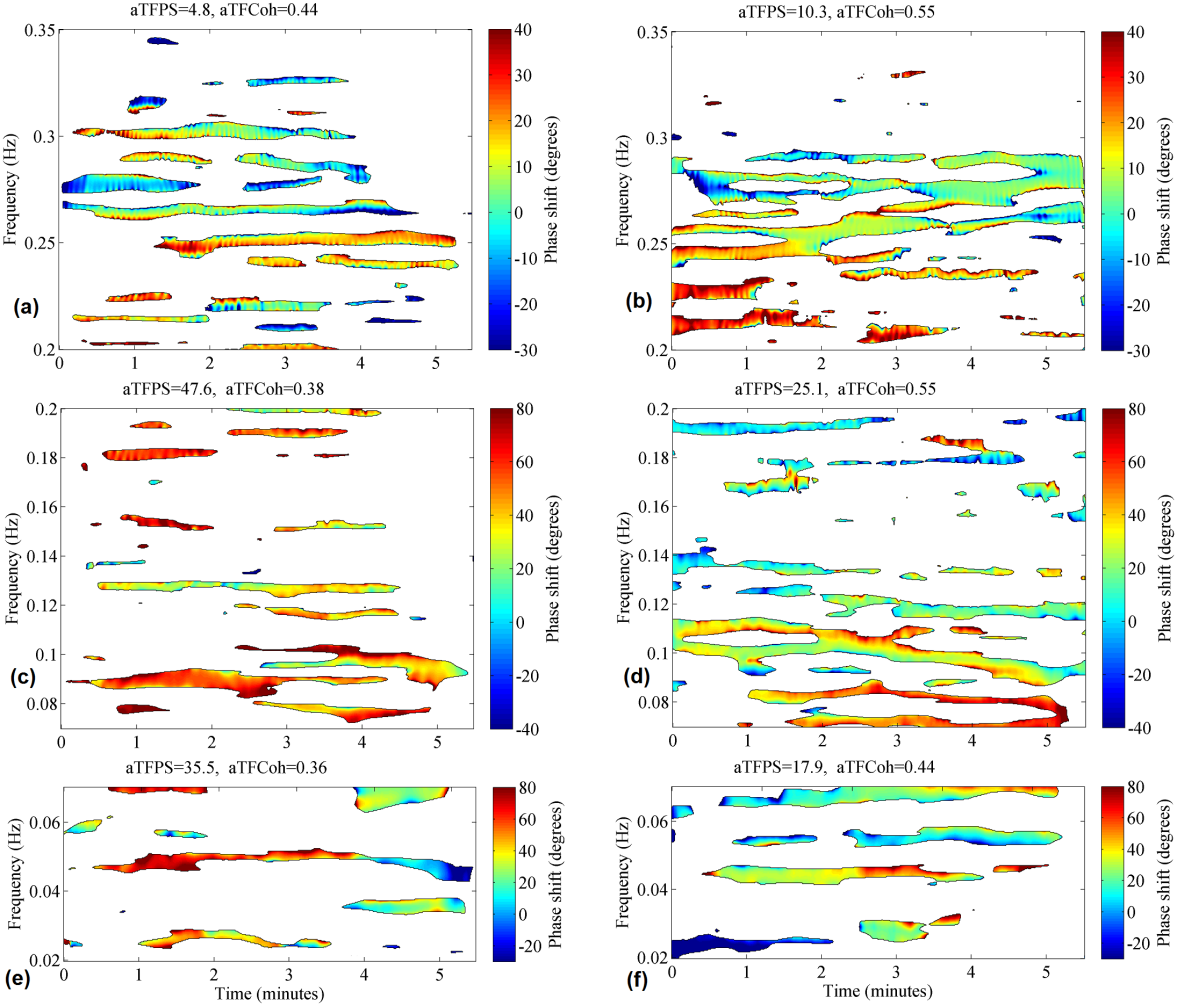
Time-frequency phase shift between arterial blood pressure and cerebral blood flow velocity for an exemplary volunteer. Plots (a), (c), (e) refers to normocapnia, whereas plots (b), (d), (f) corresponds to hypercapnia at the frequency ranges HF (0.20–0.35 Hz), LF (0.07–0.20 Hz), and VLF (0.02–0.07 Hz), respectively. Black contour confines area where magnitude-squared coherence was at least 0.9. Phase shift in the remaining area is not considered. Notation: aTFPS—average phase shift, aTFCoh—average coherence.

The median values of aTFPS, calculated for each subject within the T-F mask of TFCoh > 0.9, and their respective interquartile ranges are shown in Table 4. aTFPS in hypercapnia was significantly lower in comparison with normocapnia in the VLF and the LF but remained unchanged in the HF. Additional testing by means of Friedman ANOVA with post-hoc comparison showed that aTFPS was significantly decreased in the HF when compared with the VLF and the LF, as it was shown in Fig 6b. TFPS for a representative volunteer during normocapnia and hypercapnia in the VLF, LF, and HF are presented in Fig 7. This T-F plane shows that TFPS changes in time suggesting non-stationarity.

**Table 4:** Comparison of changes in the time-frequency phase shift during normocapnia and hypercapnia.

Parameter	Normocapnia	Hypercapnia	*p*-value
aTFPS in VLF	40.55 [30.81]	25.10 [33.85]	= 0.0005
aTFPS in LF	34.55 [16.49]	21.75 [13.89]	< 10^−7^
aTFPS in HF	5.95 [12.44]	6.49 [10.56]	= 0.22

Results are: median [IQR]. aTFPS—average time-frequency phase shift. Frequency ranges: VLF– 0.02–0.07 Hz, LF– 0.07–0.20 Hz, HF– 0.20–0.35 Hz.

### Spectral coherence and phase shift results

Average spectral coherence (aSCoh) was lower than 0.38 mostly in the VLF (Fig 6c). We found 18 cases of low aSCoh in normocapnia and 10 cases in hypercapnia. In the LF 4 cases of aSCoh < 0.38 in normocapnia and 6 in hypercapnia were observed. Similarly in the HF 8 cases of low aSCoh were found in normocapnia and 3 cases in hypercapnia.

Table 5 gives the median values and the corresponding interquartile ranges of SPS calculated at maximal value of SCoh during normocapnia and hypercapnia in the VLF, LF, and HF. In the VLF and the LF, SPS was significantly lower in hypercapnia, whereas in the HF, SPS was higher in hypercapnia when compared with normocapnia (Fig 6d). There were also significant differences between frequency ranges. SPS, like aTFPS, was significantly decreased in the HF when compared with the VLF and the LF.

**Table 5:** Comparison of changes in the spectral phase shift during normocapnia vs. hypercapnia.

Parameter	Normocapnia	Hypercapnia	*p*-value
SPS in VLF	51.32 [36.87]	23.65 [32.44]	< 10^−5^
SPS in LF	36.15 [28.69]	19.33 [12.06]	< 10^−6^
SPS in HF	6.15 [11.08]	13.43 [12.50]	= 0.017

Results are: median [IQR]. SPS—spectral phase shift calculated at maximal value of spectral coherence. Frequency ranges: VLF– 0.02–0.07 Hz, LF– 0.07–0.20 Hz, HF– 0.20–0.35 Hz.

### Comparison between time-frequency and spectral estimates of phase shift

**Fig 8:**
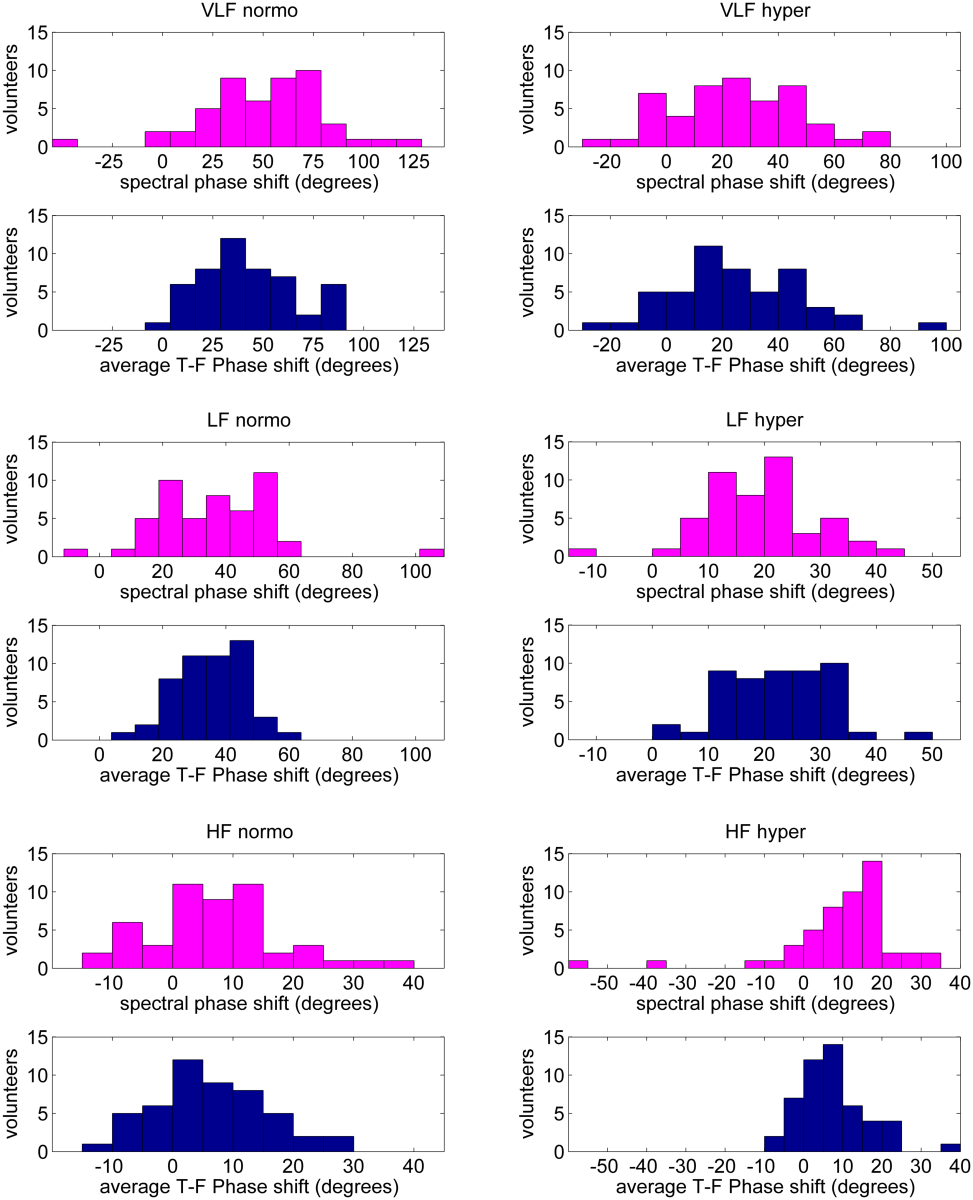
Histograms of phase shift estimates in study sample obtained using spectral method (in magenta) and time-frequency approach (in dark blue). Left column—normocapnia, right column—hypercapnia. Upper rows—VLF range (0.02–0.07 Hz), middle rows—LF range (0.07–0.20 Hz), bottom rows—HF range (0.20–0.35 Hz). Larger dispersion of spectral estimates of phase shift can be noticed in all frequency ranges.

No significant differences were observed between median values of aTFPS and SPS during normocapnia in the LF and the HF nor during hypercapnia in the VLF. However, aTFPS was lower than SPS during normocapnia in the VLF (*p* = 0.009) and during hypercapnia in the HF (*p* = 0.0012) but in the LF the opposite relation was observed (*p* = 0.007). Fig 8 shows the distributions of the phase shift estimated using T-F and spectral techniques in particular frequency ranges during normo- as well as hypercapnia. Significantly reduced variance of T-F phase estimates with respect to spectral ones was found in normocapnia in the VLF (*p* = 0.023) and the LF (*p* < 0.0001) and in hypercapnia in the HF (*p* < 10^−6^).

## Discussion

### Non-stationarity

In this paper we presented an alternative method for dynamic CA evaluation that accounts for non-stationarity of CA and the signals used for its assessment. The hypothesis of stationarity for the considered physiological signals was rejected in most cases both in normocapnia and hypercapnia (see Table 1). To the best of our knowledge spontaneous fluctuations of ABP and CBFV were not tested for stationarity before. However, the stationarity of changes in ABP and CBFV evoked by thigh cuff maneuvers were tested previously using a two-way ANOVA of the mean and standard deviation of the signals [45]. We also tested the phase shift between ABP and CBFV for stationarity itself. Stationarity of the phase shift was rejected in most cases (Table 1), however, the rate of this rejection varied across the states (normocapnia vs. hypercapnia). The rejection rate was lower during hypercapnia suggesting that disturbed CA led to the relationship between ABP and FV becoming a more stationary process.

The T-F approach allows observing periods when the local coherence is either high or low, while in the classic approach no information about temporal changes in coherence is available. A seemingly average coherence derived from Fourier methods may in fact hide its dynamic nature indicating possible periods of very high coherence that could be relevant to better understanding of dynamic CA.

### Coherence and phase shift estimation

Although the parameters used in our study give a rather basic description of dynamic CA, their interpretation is easy and widely accepted in clinical research [9,46]. Our approach combines non-stationary signal processing with localization of T-F regions of statistically justified local coupling and phase shift estimation to provide a comprehensive characterization of dynamic CA. Phase shift does not have physical sense if there is a lack of linear relation between two signals. High coherence (close to 1) reveals linear relation between two signals. However, low coherence does not prove the lack of linear relation, because co-variants that were not accounted for or noise may diminish coherence as well [12–14]. Non-linearity is present particularly in the VLF due to changing cerebrovascular resistance but the phase shift can be still useful to detect impairment of CA [47]. However, it can be shown analytically that low value of coherence leads to inflation of variance of the phase shift estimator [48]. For that reason, some studies based on transfer function exclude data affected by low average coherence from evaluating the phase shift [16–18].

In our framework phase shift between oscillations in ABP and CBFV was taken into account only in those regions where linear model was locally validated by statistically justified value of TFCoh (i.e., in regions of TFCoh > 0.9). The threshold 0.9 for significant coherence in our T-F method may look very high, particularly, when reader is contrasting it to thresholds observed in transfer function analysis based on Welch’s method. The feature which differentiates T-F transform presented in the manuscript from transfer function analysis is that data cannot be divided into segments to average spectral estimates. Such segmentation allows reducing the threshold for significant coherence. However, this is done at the expense of losing temporal resolution. Moreover, segmentation and averaging proposed by Welch is proved to improve spectral estimates assuming stationarity. Presented T-F method is not strictly equivalent to the classical transfer function approach without multi-segment averaging. Spectral coherence would be identically equal to 1.0 if only one segment was used in classical transfer function analysis. However, due to the properties of ZAMD-based method such a problem does not occur. In both classical and T-F method, the threshold was defined as the 95^th^ percentile of the distribution consisting of coherence values between 10,000 realizations of two random sequences. Depending on the considered method, we refer to TFCoh for selected points in T-F plane or spectral coherence for chosen frequencies. Very high values of spectral coherence between random sequences are extremely rare. Observing spectral coherence close to 1.0 would mean that for a given frequency two random sequences are coherent unceasingly which is unlikely in the case of sufficiently long duration of generated noise. It is, however, possible that two random sequences happen to be coherent at some point in time-frequency plane. Such cases pull the threshold for TFCoh towards higher values. The threshold for significant time-frequency coherence can be reduced by applying kernels introducing stronger smoothing of TFRs and TFCoh estimates; however, stronger smoothing implies lower T-F resolution. Nevertheless, adopting the threshold of 0.9 in T-F method used in our study did not lead to data rejection. We observed that each subject had some areas of significantly high coherence (above the 0.9 threshold) in each of the analyzed frequency ranges, i.e. VLF, LF, and HF. These areas allowed calculating representative estimate of phase shift in each case.

On the other hand, rejection of cases where average spectral coherence aSCoh did not reach the significant value of 0.38 would lead to reduction of sample size by 46%. Such an approach may introduce a bias to statistical analysis. Furthermore, it shows how problematic it can be to use spectral method in clinical studies, when only reduced amounts of data would satisfy the coherence criterion. Moreover, the fact that data might only be accepted in certain frequency ranges and not in others will make it almost impossible to compare patients with controls in a standardized manner. To avoid data rejection, it has been proposed that spectral phase shift SPS should be calculated for the frequency where coherence is maximal [44]. This approach, however, suffers from higher dispersion, since SPS is then calculated only for one frequency and does not benefit from variance reducing averaging.

### The issue of phase wraps

In our cohort, the wrap around of phase estimates was often associated with low coherence. Visual inspection of each TFPS representation within the T-F mask of TFCoh > 0.9 revealed 6 cases of wraps in normocapnia and 3 in hypercapnia in the VLF. In the LF there was only one case in normocapnia, and the HF was free of wraps. However, majority of studies based on transfer function do not report how the phase wrap was managed. Tzeng *et al*. [49] did not implement any procedure of handling phase wraps. Data affected by phase wrapping can be simply eliminated from further analysis but it leads to data loss [50]. However, presented T-F method allows identifying regions containing both very low and very high values of phase that indicate wraps. Therefore, such the regions were excluded from calculating mean values of phase without the necessity of the whole recording rejection and reducing the size of study sample. Although similar procedure can be applied in transfer function analysis, it leads to excluding entire frequencies affected by wraps instead of T-F regions and hence more information are lost. Estimation of TFPS within T-F areas at high coherence decreases phase wrap around effect and can reduce phase dispersion when compared with a spectral approach. Furthermore, averaging aTFPS metrics diminishes the risk that the result is affected by some extreme value, which is likely to happen when SPS is evaluated for a single frequency.

### Interpretation of the results

It was not our main goal to study the influence of hypercapnia on CA, effect that has been known for many years. On average, the TFCoh in normocapnia was weak. This confirms the findings reported by numerous studies where spectral coherence was used [13,14,24]. However, the absolute values of spectral coherence and T-F coherence cannot be compared as they are calculated using different estimation techniques [39].

In traditional Fourier-transform-based analysis the spectral coherence is assumed to be constant for relatively long time and its low value leads to rejection of estimates of phase between CBFV and ABP particularly at low frequencies where dynamic CA is expected to be the most efficient. The ZAMD-based TFCoh estimates were not constant during the observation time of 5 minutes which seem to suggest that dynamic CA is a time-variant mechanism, even at these short time scales. However, it could also reflect non-stationary character of exogenous factors of ABP—CBFV model. We found that in normocapnia about 9% of TFCoh in the VLF was > 0.9 and this percentage of statistically justified coherence became higher in the LF (about 14%) and the HF (about 12%).

In our study, hypercapnia, which was used as a model of CA impairment, led to an increase of TFCoh particularly in the LF and the HF and to a decrease in TFPS in the VLF and the LF but not in the HF. It is in line with the previous studies where spectral coherence and phase shift were used [8,11]. Impaired CA allows changes in ABP to be passively followed by changes in CBFV hence the value of coherence becomes higher [11] and phase shift becomes lower [8]. Our results indicate that the TFPS has higher discrimination rate in the context of *p*-value for the VLF than that of TFCoh, while for the LF both phase and coherence achieve similar discrimination rate (see Tables 3 and 4). It was recently reported that phase shift is reduced in stroke patients when compared with normal subjects in wide frequency range 0.02–0.38 Hz. Those results suggest that CA might be active in higher frequencies corresponding to respiratory oscillation as well [30]. The method used in that study accounts for nonlinearity of pressure-flow relationship and no assumption of high coherence was done for phase shift estimation in that approach.

The classic method, unlike the TF approach, provided higher values of phase shift in the HF range during hypercapnia, whereas one could expect the lower values when CA mechanism is impaired [30]. However, only weak correlation between spectral phase shift calculated from respiratory oscillations and CA has been reported by the other studies [50,51]. Furthermore, decreased respiratory rate during hypercapnia may be the cause of increased values of spectral phase shift in the HF range as the negative relationship between the two of them has already been shown [50] and also confirmed in our data (*R*_Spearman_ = −0.32, *p* = 0.022). This may suggest that spectral phase shift calculated in the HF range is modulated by respiratory rate and reflects changes in CA to a lesser extent than phase shift calculated in VLF and LF ranges. No changes in TFPS in HF range found in our study may in turn suggest that our approach is sensitive to both decreased respiratory rate and CA derangement caused by hypercapnia but their impacts on TFPS are mutually abolished.

In our data, it may appear that the classic Fourier-based method arrives at slightly better discrimination rates than the T-F method. This is only the case for the HF and the VLF range (lower *p*-value). However, the former may not assess the changes in CA as it was discussed above, and as far as the latter is concerned, the T-F method was characterized generally with lower variance of the phase estimator than the classic method.

Significantly reduced dispersion of T-F phase estimates with respect to spectral ones was found during normocapnia in the VLF and the LF and also during hypercapnia in the HF. This reduced dispersion in normocapnia was most likely due to lower associated coherence values, which translated into increased variance of the phase estimator [48]. The main advantage of the presented T-F method is that it extracts regions of high coherence in T-F plane for the calculation of phase shift and thus tends to be more resilient to the influence of extraneous noise or non-linear effects than classic approach.

The results presented in Fig 6 demonstrate the frequency-dependent behavior of dynamic CA, which was also shown in previous studies using transfer function analysis [11] and nonlinear dynamic approach [30].

### Comparison to other methods

In this study T-F analysis was performed using ZAMD distribution belonging to the Cohen class, as it allows to achieve high resolution both in frequency and time domain while keeping cross-terms relatively reduced [32]. There have already been a number of studies on CA using wavelet-based approaches [52–54], autoregressive moving-average (ARMA) [55–57], recursive least squares [58,59], and Volterra series [25–27].

In contrast to other methods, using wavelets [52–54] as well as ZAMD-based algorithm to estimate CA index does not require identification of input-output model of CA. One of the crucial aspects of wavelet analysis is the selection of a proper orthogonal family, since it has a substantial influence on the final multistage decomposition. Unlike methods from the Cohen class which lead directly to time-frequency domain, wavelet transform decomposes signal into time-scale domain. The interpretation of wavelet representation in time domain is similar to TFR, but retrieving the frequency is often more difficult.

ARMA-based models have been used to track CA, among others, during hypocapnia [55], hypercapnia [55,56], and Valsava maneuver [57]. The advantage of that approach is that it requires only a relatively short data window (30–60 s) which can be moved by about 1 s at a time. Upon identification of ARMA model for each data segment, time-varying autoregulation index (ARI) can be obtained by fitting CBFV/ABP step responses to template step responses [60]. That method has better time resolution than ZAMD-based approach, since TFR needs to be smoothed to eliminate interferences characteristic to bilinear T-F distributions. However, ARI, unlike the phase shift, cannot be used directly to study frequency-dependent behavior of CA. Moreover, it is a parametric method that assumes certain second order model of CA and ignores any noise/non-linear effects contributions.

CA has been also modeled using recursive least squares adaptive filter [58,59] with Hilbert transform employed to calculate instantaneous phase shift between ABP and CBFV [59]. To focus the analysis on a particular frequency band, corresponding band pass filter should be applied prior to the system identification and performing transform. Therefore, it requires repeating the entire procedure each time a different frequency band is analyzed. As far as T-F methods from Cohen class and wavelet transform are concerned, one needs to calculate transform only once to obtain the entire spectrum, even though narrow frequency band may be demanded. Liu *et al*. [59] estimated Hilbert-transform-derived instantaneous phase shift for the entire duration of the recording. In our case individual T-F phase spectra were fragmented due to the requirement of analyzing phase only when coherence is above the considered threshold. In order to extract time-varying phase shift for given frequency for each recording, this requirement could be relaxed, however, the phase estimator has larger variance when corresponding coherence is low [48].

CA has been also modeled using Volterra series as a system consisting of linear and nonlinear components [25–27]. This approach allows investigating non-stationarity of linear and nonlinear components related to ABP as well as EtCO_2_. Even though nonlinear components contribute to improve model quality, it is difficult to generate physically-interpretable CA parameter like phase shift or ARI [15,24,28]. Moreover, increasing accuracy of Volterra models usually requires an exponential growth of number of necessary Volterra kernels making it computationally challenging.

### Limitations

We have to admit that T-F approach was not as beneficial as it could be expected. This was perhaps because the method did not include the inherently nonlinear properties of CA, and thus suffer from the same problems as the classic spectral approach. The proposed approach is limited to linear interaction between oscillations in ABP and CBFV. Although in general CA is described by a nonlinear ABP-CBFV relationship [13], it is often assumed that amplitudes of ABP slow waves are sufficiently small and the analysis period sufficiently short to justify neglecting the non-linear terms. The transfer-function-based parameters such as: coherence or phase shift have straightforward physical meaning and are widely used in clinical investigations to quantify dynamic CA [11,16,18]. It has been suggested that regulation of CBF is better modeled using multivariate models that include both ABP and EtCO_2_ as inputs [26–28,61,62]. Our linear models did not take into account potential interactions between EtCO_2_ and ABP which are taken into consideration in nonlinear models [26,27,61]. However, we selected steady state segments of data corresponding to normo- and hypercapnia where EtCO_2_ was sufficiently stable during the analysis period. Next, we did not investigate transitional phase of increasing EtCO_2_ [27,58,62]. Presented T-F method takes into account the issue of non-stationarity, however, the benefit from applying T-F analysis was relatively small.

There exist measures such as partial [63,64] and multiple coherence [10,28] that can be used to address the question of influence of arterial tension of CO_2_ on CBFV variations and they could be extended for the nonstationary case. Our unpublished results showed, that partial TFCoh was generally lower than univariate TFCoh in all three frequency ranges. Estimating TFPS in areas of high partial TFCoh, however, neither influenced its value nor its dispersion. Multiple coherence, on the other hand, mainly may answer whether assumed linear multivariate system can describe the CBFV variability, particularly in low frequency range [28], but does not extract directly regions of meaningful phase shift between ABP and CBFV oscillations.

We used Transcranial Doppler blood flow velocity as a surrogate of CBF. This holds as long as the diameter of insonated artery remains constant. Previous studies performed in the presence of large alteration in EtCO_2_ reported that significant changes in the diameter of middle cerebral artery are rather unlikely [65,66]. We measured ABP from finger and CBFV from middle cerebral artery. This difference in location of measurement my cause a time delay between CBFV and ABP recordings and contribute to overestimation of the phase shift at high frequencies (> 0.38 Hz) [30]. To ensure reliable estimates of ABP-CBFV we studied the phase shift in the frequency range of 0.02–0.35 Hz.

## Conclusions

We presented an alternative method to study dynamic CA. The proposed approach accounts for non-stationarity of CA as well as the analyzed signals and provides estimates of phase shift between arterial blood pressure and cerebral blood flow velocity oscillations in time-frequency regions of statistically justified value of coherence. Our analysis of non-stationary character of CA did not in the end offer significant benefits for short recordings based assessment over traditional spectral approach. That fact, on the one hand, strengthens the validity of the classic transfer function based approach. On the other hand, T-F method performed no worse than the stationary one and yet may offer benefits from lower dispersion of phase shift as well as a more in-depth insight into the dynamic nature of CA.

## Supporting information

S1 Dataset Signals database. Database of physiological signals registered in 50 volunteers during normocapnia and hypercapnia.
(ZIP)
